# Short-Term Outcomes of Sleeve Gastrectomy for Morbid Obesity: Does Staple Line Reinforcement Matter?

**DOI:** 10.1007/s11695-014-1251-9

**Published:** 2014-05-09

**Authors:** Ertugrul Kemal Durmush, Goktug Ermerak, Deniz Durmush

**Affiliations:** The Life Weight Loss Centre, Level 4, 171 Bigge Street, Liverpool, NSW 2170 Australia

**Keywords:** Obesity, Sleeve gastrectomy, Staple line leak, Staple line reinforcement, Polyglycolic acid/trimethylene carbonate, Bioabsorbable

## Abstract

**Background:**

Stand-alone laparoscopic sleeve gastrectomy (LSG) has been found to be effective in producing weight loss but few large, one-center LSG series have been reported. Gastric leakage from the staple line is a life-threatening complication of LSG, but there is controversy about whether buttressing the staple line with a reinforcement material will reduce leaks. We describe a single-center, 518-patient series of LSG procedures in which a synthetic buttressing material (GORE® SEAMGUARD® Bioabsorbable Staple Line Reinforcement) was used in the most recently treated patients.

**Methods:**

We retrospectively reviewed the medical records of all patients who underwent LSG in our unit between September 2007 and December 2011. Patients treated before August 2009 did not receive the staple line reinforcement material (*n* = 186), whereas all patients treated afterward did (*n* = 332).

**Results:**

The percentages of excess weight loss in the 518 patients (mean age, 41 years; 82 % female; mean preoperative body mass index, 44 kg/m^2^) were 67 % (79 % follow-up rate) at 6 months postoperatively, 81 % (64 %) at 1 year, and 84 % (30 %) at 2 years. Type 2 diabetes resolved in 71 % of patients (91/128). Patients given reinforcement material had baseline characteristics similar to those in the no-reinforcement-material group, but had no postoperative staple line leaks or bleeding. The no-reinforcement group had three leaks (*p* = 0.045) and one case of bleeding.

**Conclusions:**

LSG resulted in substantial short-term weight loss. Use of the bioabsorbable staple line reinforcement material may decrease leaks after LSG.

## Introduction

Laparoscopic sleeve gastrectomy (LSG) is a treatment for obesity that involves removal of the fundus and most of the antrum of the stomach, thereby creating a gastric tube or sleeve that restricts oral intake. Several recent randomized studies [1–5], nonrandomized comparison investigations [6–9], and analyses of registries [10, 11] and large series [12, 13] have found that use of LSG as a stand-alone procedure is safe and effective in achieving weight loss. Postsurgical resolution of comorbid conditions such as type 2 diabetes mellitus (T2DM) and hypertension have also been reported [2, 14–20]. Advantages of LSG over other types of bariatric surgery include its relative technical simplicity, preservation of the pylorus, and avoidance of postoperative malabsorption [3, 14, 21]. The popularity of LSG among surgeons and patients continues to increase.

The reported percentage of excess weight loss (%EWL) after LSG has varied widely, partly because of differences in the duration of follow-up and number of patients followed. A 2012 systematic review of 123 articles (covering 12,129 patients) found that mean %EWL is about 60 % (range, 30–83 %) at 1 year after surgery and 65 % (range, 46–75 %) at 2 years [22]. An assessment of questionnaire responses (covering 19,605 procedures) by the Third International Summit on LSG yielded similar results, with mean %EWL values of 63 and 65 %, respectively, at 1 and 2 years postoperatively [21].

Although bariatric surgery is considered safe, it has risks, with the most serious being gastric leakage from the staple line. Possible sequelae of a staple line leak include abdominal sepsis, chronic gastric fistula, multiorgan failure, and death [23]. The leading cause of death after LSG is staple line leakage [23]. Fortunately, leak rates are low, with commonly reported mean values ranging from 1 to 3 % [1, 11, 19, 23–26], although rates up to 10 % have been observed [25]. Bleeding from the staple line can also occur after LSG, in perhaps 1 to 2 % of patients [4, 5]. This complication is usually less serious than a staple line leak, but it can result in financial costs and patient discomfort associated with blood transfusion or re-exploration [27].

Reinforcement of the staple line has been proposed as a method for preventing leaks and bleeding after LSG. Methods of reinforcement include oversewing the staple line, applying a fibrin sealant, and using a buttressing material. Several buttressing materials are available, but the most widely used are a synthetic bioabsorbable material composed of the copolymer polyglycolic acid/trimethylene carbonate (PGA/TMC) (GORE® SEAMGUARD® Bioabsorbable Staple Line Reinforcement, W.L. Gore & Associates, Elkton, MD, USA) and bovine pericardium. A recent meta-analysis found that for 55 publications (covering 6,578 patients) in which staple line buttressing practices in LSG were described, some kind of buttressing material was used in 82 % of cases and bioabsorbable buttressing material was employed in 56 % [26]. The meta-analysis also observed that use of a bioabsorbable buttressing material reduced the leak rate from 3.2 to 2 %, although the decrease was not statistically significant. In addition, three small randomized clinical trials (*n* = 40 [28], 25 [29], and 40 [30], respectively, in each treatment arm) found no significant difference in leak rate between patients given the PGA/TMC reinforcement material and those in whom either oversewing [28–30], bovine pericardium [30], or no reinforcement [29] was used. However, the PGA/TMC group had a 0 % leak rate in one of those studies [30] and a significantly lower amount of blood loss (*p* = 0.03) in another [29].

In contrast to the results of these trials, a meta-analysis by Choi et al. [31] (covering 1,335 patients) and a 230-patient series by Daskalakis et al. [32] showed that use of staple line reinforcement significantly decreased the rate of postoperative leaks, hemorrhage, or both. Moreover, in a nonrandomized comparison study (118 total patients), Ser et al. [33] found that staple line reinforcement significantly reduced postoperative leaks (to 0 %) compared with no reinforcement (*p* = 0.004). Saul et al. [34] reported no leaks with use of the PGA/TMC reinforcement material in an LSG series at a Veterans Affairs medical center. Finally, an evaluation of the Spanish National Registry of 540 LSG procedures found that patients in whom staple line reinforcement was used had a significantly lower rate of postoperative complications (*p* = 0.039) and a leak rate which was half that in patients given no reinforcement (2.6 vs 5.3 %) [10].

Aside from staple line leaks and bleeding, issues pertaining to LSG that have been the focus of many research efforts include the optimal bougie size and distance from the pylorus at which gastric division should begin. The Spanish registry data indicated that use of a smaller bougie (32 F–36 F) was associated with better weight-loss outcomes at 1 year postoperatively, without an increase in complications [10]. In their meta-analysis, Parikh et al. [26] found no significant difference between bougies smaller than 40 F and those 40 F or larger with respect to weight loss up to 1 year, but they did observe a significantly higher leak rate with smaller bougies (*p* = 0.0009) that was presumably related to an increase in intraluminal pressure. Gagner [35] noted that avoiding leaks depends on using a bougie of at least 50 F.

The evaluation of the Spanish registry data found that beginning the gastrectomy close to the pylorus resulted in better weight loss [10]. Bellanger and Greenway [13] commented that starting the resection 3 to 4 cm from the pylorus decreases the antral volume while preserving its function, thereby reducing the risk of distal stricture and proximal leaks. Others prefer to begin the resection more than 4 cm from the pylorus with the aim of preserving the gastric antrum and improving gastric emptying [36].

The variations in results of studies addressing staple line reinforcement, bougie size, and distance from the pylorus in LSG indicate clearly that more research on the technical aspects of the procedure is required. We describe a large (518 patients), one-center study designed to obtain new data on these issues.

## Methods

### Study Design

All patients provided informed consent to treatment. Data was acquired from a retrospective review of the records of patients who underwent LSG at our center between September 2007 and December 2011. Preoperative patient characteristics (age, sex, weight, body mass index [BMI], comorbid conditions, and previous bariatric surgery); operative factors (operating time, concomitant procedures, and bougie size); and intraoperative and postoperative adverse events, including staple line leaks, were recorded. Patients who were lost to follow-up were included only in the assessments of surgical morbidity and immediate postoperative complications, not in the weight-loss analyses.

Before their LSG procedure, patients were advised to quit smoking and to consume a very-low-calorie diet for a period ranging from 1 week to 2 months. Patients were also expected to lose about 10 % of excess weight preoperatively.

Staple line reinforcement material was not used in the 186 patients who underwent surgery before August 2009 (no-reinforcement-material group) because it was unavailable. In August 2009, as part of our ongoing efforts to prevent leaks by exploring promising new surgical techniques, we began to use the bioabsorbable PGA/TMC material to buttress the staple line in all patients (reinforcement-material group; 332 patients).

All patients were scheduled to return for a follow-up visit with their surgeon 3 months, 6 months, 1 year, 1.5 years, and 2 years postoperatively. Follow-up evaluations included calculation of %EWL and assessments to determine whether T2DM and hypertension had resolved or improved in patients who had these conditions preoperatively. The %EWL was calculated as follows: (preoperative weight minus postoperative weight) / preoperative excess weight times 100. Preoperative excess weight was calculated as follows: preoperative weight minus ideal weight (based on a BMI of 25). Fourteen percent of the follow-up evaluations for %EWL were conducted by telephone. An improvement in hypertension or T2DM was defined as a reduction in the dosage or number of medications taken to control these conditions; a resolution of hypertension or T2DM was defined as complete discontinuation of all therapeutic agents with maintenance of normoglycaemia.

### Surgical Technique

All LSG operations were accomplished laparoscopically; no conversions to an open procedure were required. An optical trocar was placed in the left upper quadrant. A 5-mm port was inserted in the left subcostal area; a 15-mm port was placed in the right side of the abdomen, in the midclavicular line; and a 12-mm port was inserted in the epigastrium. A Nathanson retractor was used to lift the left lobe of the liver to provide full access to the upper stomach. The short gastric vessels were divided with a Harmonic scalpel (Ethicon Endo-Surgery, Cincinnati, OH, USA). The dissection was done from the pyloric region to the left crus. The left crus was fully dissected until the right crus became visible to allow identification of any hiatal hernia (HH). A bougie was then inserted to gauge the new stomach. The first two firings of the Echelon 60 Endopath stapler (Ethicon Endo-Surgery) were done from the port on the right side; the remaining firings were done through the epigastric port, toward the gastroesophageal junction (GEJ) but keeping slightly away from it in order to avoid injury to the cardia.

Gastric resections were begun 2 to 4 cm from the pylorus. In the early part of the series (no-reinforcement-material group), only 40 F bougies were used. Subsequently (reinforcement-material group), the bougie size ranged from 32 F to 40 F, but a 36 F bougie was used in almost half of the operations (174). Stapling was done with green reloads. In patients in whom the bioabsorbable PGA/TMC material was not used, either oversewing or both oversewing and fibrin sealant (Tisseel, Baxter, Deerfield, IL, USA) were applied to the staple line. In patients given the PGA/TMC material, “figure-of-eight” stitches were placed at intersections so that the entire staple line became one unit. Moreover, in all patients, the gastrosplenic ligament was attached to the new stomach by placing sutures at the intersections of the staple line with the aim of further securing the staple line and preventing “spiraling” of the new stomach. Any HH found was repaired by placement of nonabsorbable monofilament polybutester sutures (Novafil, Covidien, Mansfield, MA, USA) either posteriorly, anteriorly, or both. Novafil sutures were also used to repair the phrenogastric ligament if it had been divided during the dissection. After hemostasis was achieved, fibrin sealant was sprayed on the staple line. In most cases, a Blake drain was inserted and removed on the second postoperative day.

In the first 186 patients in the series (no-reinforcement-material group), a methylene blue leak test was performed before discharge from the hospital. In the reinforcement-material group, an intraoperative leak test using either methylene blue or the air-bubble method was initially performed routinely. However, testing for leaks intraoperatively and in the immediate postoperative period was subsequently discontinued because of the poor yield of the assessments: all staple line leaks in the series occurred after hospital discharge. In addition, many patients had pain at the drain site or in their left shoulder, and this was observed to subside immediately after drain removal.

### Postoperative Care

Patients received morphine only on the day of surgery; subsequently, intravenous paracetamol and indomethacin suppositories were used for pain relief. Intravenous antibiotics were administered prophylactically for 2 days after surgery, and intravenous fluids were continued until hospital discharge. Only patients who had obstructive sleep apnea or a serious medical problem were admitted to the intensive care unit for close monitoring (for one night) postoperatively.

Patients began to consume a clear-fluid diet the day after surgery and progressed to a full-fluid diet before discharge. They continued the full-fluid diet until their visit with a dietitian 2 weeks postoperatively, after which they switched to a mashed/blended diet for about 2 weeks. Most patients started to consume solid food by 5 or 6 weeks after LSG. They visited the dietitian again at this time and were encouraged to eat five or six small, protein-rich meals a day and to drink low-calorie liquids between meals. In addition, administration of a proton-pump inhibitor was begun and continued for 6 months and lifelong multivitamin supplementation was prescribed.

Patients were also seen by an exercise physiologist before surgery and 5 weeks afterward to establish an exercise plan. This plan and the patients’ compliance with it were subsequently reviewed as needed. Moreover, a psychology consultation was obtained before surgery to assess patients’ psychological readiness for LSG, and postoperative follow-up visits were made on an as-needed basis.

### Data Analysis

Data were entered into a database and compiled. Results are presented as either numbers and percentages or means ± SD. Comparisons between the no-reinforcement-material group and the reinforcement-material group were done by using *t* test or Fisher exact test. A *p* value of less than 0.05 was considered to represent a significant difference between groups. All statistical analyses used JMP software (version 8.0.1, SAS Institute, Cary, NC, USA).

## Results

Table 1 shows baseline characteristics of the 518 patients who underwent LSG. Adverse events that occurred perioperatively or during follow-up are shown in Table 2. The overall adverse-event rate was 1.7 %; the staple line leak rate was 0.6 %. One patient had onset of gastroesophageal reflux disease (GERD) with persistent belching. When conservative treatment did not resolve this problem, the patient underwent gastric bypass, which was successful. All three staple line leaks in the series were observed on the sixth postoperative day. Two leaks were at the GEJ; the other was near the antrum. One of the patients in whom a leak occurred died (0.2 % series mortality rate) of sepsis and multiple organ failure 9 months following LSG, after six laparotomies, stent placement, and use of total parenteral nutrition failed to resolve the leak. The other two patients with a staple line leak underwent stent placement, and the leaks resolved at 6 weeks and 5 months, respectively, after LSG.

**Table 1:** Baseline characteristics of 518 patients who underwent LSG

Characteristic	Value
Mean ± SD age (range), years	40.9 ± 10.7 (17–65)
Female/male, *n* (%)	425 (82)/93 (18)
Mean ± SD preoperative weight (range), kg	118.4 ± 24.1 (73–214)
Mean ± SD preoperative BMI (range), kg/m^2^	43.9 ± 7.6 (30–78)
Type 2 diabetes mellitus, *n* (%)	128 (25)
Hypertension, *n* (%)	165 (32)
Previous bariatric surgery, *n* (%)	19 (4)

*LSG* laparoscopic sleeve gastrectomy, *BMI* body mass index

**Table 2:** Adverse events after LSG in 518 patients

Complication	Number (%)
Death^a^	1 (0.2)
Staple line leak	3 (0.6)
Partial splenic infarction	1 (0.2)
Postoperative bleeding	1 (0.2)
Infected hematoma	2 (0.4)
Leak through broken gastropexy stitch	1 (0.2)

*LSG* laparoscopic sleeve gastrectomy

^a^Caused by sepsis and multiple-organ failure 9 months following LSG and after several unsuccessful attempts to resolve a staple line leak

One patient in the series had a leak that was not at the staple line but originated from a gastropexy stitch placed to secure the stomach and prevent sliding (the patient had a large HH). The suture broke and a small fistula formed. A laparoscopic examination performed 7 days postoperatively revealed the leak, after computed tomographic scanning failed to detect it. The leak was treated by performing a suture repair covered with an omental patch and sprayed with fibrin glue, but it recurred in 4 days. The patient then underwent laparoscopy, washout, and placement of additional drains around the leak site, resolving by about 5 months.

Data on weight loss were collected from 79 % of the patients (*n* = 409) in the series at 6 months postoperatively, 70 % (*n* = 329) at 1 year, and 77 % (*n* = 258) at 2 years. The %EWL in the short term was 67.1 % at 6 months, 81.2 % at 1 year, and 83.8 % at 2 years. During follow-up, T2DM resolved completely in 91 of 128 patients (71 %) and improved in 23 (18 %). Hypertension resolved in 64 of 165 patients (39 %) and improved in 54 (33 %).

Table 3 shows baseline, operative, and adverse-events data in the study, according to whether the bioabsorbable staple line reinforcement material was used during LSG. There were no significant differences between the reinforcement-material group and the no-reinforcement-material group in the patients’ demographic characteristics or the overall postoperative adverse-event rate. In the no-reinforcement-material group, one patient had persistent GERD and distressing burping after LSG, one had an infected hematoma, one had bleeding requiring laparotomy, and three had a staple line leak. In the reinforcement-material group, there was one leak from a broken gastropexy stitch, one infected hematoma, one partial splenic infarction, and no staple line leaks or hemorrhages. The difference between the groups with respect to staple line leaks was significant (*p* = 0.045). The reinforcement-material group also had a significantly shorter operating time and smaller bougie size, as well as a significantly higher rate of HH repairs.

**Table 3:** Baseline, operative, and adverse-events data in patients in whom the staple line was reinforced (*n* = 332) and not reinforced (*n* = 186) with reinforcement material

Variable	Patients given material	Patients not given material	*p* value
Mean ± SD age, years	40.3 ± 11	41.8 ± 11	0.13
Female/male, *n* (%)	277 (83)/55 (17)	148 (80)/38 (20)	0.28
Mean ± SD preoperative BMI, kg/m^2^	44.5 ± 7.5	44.6 ± 7.7	0.1
Previous bariatric surgery, *n* (%)	15 (4.5)	4 (2.2)	0.22
Mean ± SD operating time, min	72.2 ± 20	85.1 ± 29.2	<0.0001
Mean ± SD bougie size, *F*	33.5 ± 1.9	40 ± 0	<0.0001
Concomitant HH repair, *n* (%)	176 (53)	34 (18)	<0.0001
Adverse events, *n* (%)	3 (0.9)	5 (3.2)	0.14
Staple line leaks, *n* (%)	0 (0)	3 (1.6)	0.045
Leak through broken gastropexy stitch, *n* (%)	1 (0.3)	0 (0)	1

*BMI* body mass index, *HH* hiatal hernia

## Discussion

In this series, we observed values for postoperative %EWL that are among the highest reported in the literature (Table 4). Moreover, complete resolution of T2DM occurred after LSG in more than two thirds of patients who had the condition preoperatively and hypertension resolved in nearly 40 %. The operative and postoperative adverse-event rates, including the rate of staple line leaks, were low. There were no staple line leaks in the 332 patients given the synthetic bioabsorbable PGA/TMC staple line reinforcement material, even though the stomach resection was begun close to the pylorus and bougies smaller than 40 F were employed. Like others [10, 13, 15], we believe that use of a smaller bougie produces greater weight loss, but we are aware that employing a small bougie may increase the risk of staple line leaks caused by an increase in intraluminal pressure, especially at the angle of His [35, 41]. However, our results provide new evidence that using the PGA/TMC reinforcement material mitigates that risk. Our findings support those of previous LSG series in which low leak rates (or no leaks) were observed in patients in whom both this material and a small bougie were used. These series include those of Gluck et al. (no leaks; 34 F) [39], Jacobs et al. (1.3 % leak rate; 36 F in 153 patients) [38], and Saul et al. (no leaks; 34 F) [34]. On the other hand, Bellanger and Greenway [13] used a 34 F bougie, applied only fibrin glue to the staple line, and had no leaks in 529 patients.

**Table 4:** Large series of laparoscopic sleeve gastrectomy (LSG) procedures

Series, year					Mean or median %EWL (%FU)				
	Patients, *n*	Pre-LSG BMI, kg/m^2^	Bougie size, *F*	Staple line leaks, *n* (%)	6 months	1 year	2 years	T2DM: %res/%imp	HTN: %res/%imp
Menenakos, 2010 [37]	261	45.2	38	10 (3.8)	41 (ND)	66 (ND)	65 (ND)	84^a^	89^a^
Srinivasa, 2010 [18]	253	50	36	6 (2.4)	ND^b^	ND^b^ (28)	ND^b^ (4)	81/9	48/29
Jacobs, 2010 [38]	197	44.7	36–46	2 (1.3)	ND	78 (83)	75 (83)	82/18	ND/ND
Bellanger, 2011 [13]	529	44.3	34	0 (0)	42 (71)	66 (68)	66 (63)	ND	ND
Gluck, 2011 [39]	204	45.7	34	0 (0)	64 (68)	68 (38)	62 (17)	71/28	68/29
Atkins, 2012 [15]	294	42.4	40 or 50	8 (2.7)	~48 (67)	~61 (78)	~68 (46)	75/ND	50/ND
Boza, 2012 [12]	773^c^	37.4	60	7 (0.7)	81 (52)	87 (45)	84 (25)	100^a^	98^a^
Kehagias, 2012 [40]	208^d^	43.2	32	12 (5.8)	ND	79 (98)	78 (89)	89^e^/ND	43^e^/ND
Durmush (current)	518	43.9	32–40	3 (0.6)	67 (79)	81 (64)	84 (30)	71/18	39/33

*%EWL* percentage excess weight loss, *FU* follow-up, *BMI* body-mass index, *T2DM* type 2 diabetes mellitus, *HTN* hypertension, *res* resolved, *imp* improved, *ND* no data

^a^Either resolution or improvement

^b^%EWL was not calculated, but 171 patients with a mean follow-up time of 1 year had a mean percentage of excess BMI loss of 59 %

^c^Women only

^d^Five procedures were open, but were not conversions from the laparoscopic approach

^e^At 1 year

Aside from having no staple line leaks, our patients who were given the bioabsorbable reinforcement material had no bleeding from the staple line. A significant reduction in bleeding with this material compared with no buttressing or oversewing the staple line during LSG was previously reported by Dapri et al. [29] and Consten et al. [27]. Moreover, the 2012 consensus statement on best practices from the International Sleeve Gastrectomy Expert Panel, which was based on experience with more than 12,000 cases, included a 100 % agreement with the consensus point, “Staple line reinforcement will reduce bleeding along the staple line” [41]. The mechanism by which use of staple line reinforcement material decreases the risk of staple line bleeding is unknown, but may be related to the compressive effect of the material on the transected tissue [27, 42].

Our results also support those of previous investigations which found that applying staple line reinforcement material significantly reduced operating time compared with the time required for oversewing [28–30]. In our series, the mean operating time was about 13 min shorter in patients given the material.

The limitations of our study include its nonrandomized, retrospective nature and its relatively short follow-up duration. However, new, sufficiently powered randomized trials comparing the effects of methods of buttressing, including no buttressing, on staple line leaks after LSG are unlikely to be performed for two principal reasons. One is that staple line leak rates are so low that perhaps as many as 10,000 patients would have to be enrolled in each treatment arm for a difference between techniques to be discerned [36]. The other is that most surgeons apparently already routinely use some type of buttress, especially bioabsorbable reinforcement material [26], during LSG, and they may therefore have concerns about withholding the material to fulfill randomization requirements.

Although the %EWL values in our series were high, a longer follow-up will be required to assess the effects of our LSG technique more definitively. Indeed, partly because stand-alone LSG is a relatively new procedure, there remains a general paucity of solid long-term data on its effectiveness with respect to weight loss, alleviation of comorbid conditions, and the need for additional obesity therapy, as well as its possible association with undesirable postoperative occurrences such as the persistence or onset of GERD. Overall, the numbers of patients followed for long periods have been low, and some discrepancies in findings have appeared. For example, Eid et al. [43] found good maintenance of weight loss in 69 patients followed for 3 to 8 years, whereas D’Hondt et al. [14] observed a tendency to regain weight in 23 patients followed for 6 years. Himpens et al. [44] reported weight regain and new cases of GERD in the interval 3 and 6 years after LSG in 30 patients with long-term follow-up. Kehagias et al. [40] and Bohdjalian et al. [45] noted a decline in %EWL weight loss to about 56 % at 5 years postoperatively in 27 and 26 patients, respectively, but considered these results satisfactory for their patient populations. Jiménez et al. [17] found that the T2DM recurrence rate (after initial remission) was 16 % in about 50 patients followed for 2 to 5 years after LSG; not surprisingly, recurrence was associated with a low %EWL.

## Conclusions

In a 518-patient series, LSG performed by using a bougie that was 40 F or smaller and limiting the antrum size to 2 to 4 cm resulted in excellent short-term %EWL results at 6 months and 1 and 2 years after surgery, with few adverse events. In addition, resolution of or improvement in T2DM and hypertension occurred in 89 and 72 % patients, respectively. Patients in whom synthetic PGA/TMC staple line reinforcement material was applied during LSG had no postoperative leaks or hemorrhages from the staple line. The difference in leak rate between the reinforcement-material group and the no-reinforcement-material group was significant (*p* = 0.045). Long-term follow-up in large series can provide important data on sustained weight loss and other health outcomes in patients who have undergone LSG.
